# High Frequency of Post-Transfusion Microchimerism Among Multi-Transfused Beta-Thalassemic Patients

**DOI:** 10.3389/fmed.2022.845490

**Published:** 2022-02-16

**Authors:** Spyridon Matsagos, Evgenia Verigou, Alexandra Kourakli, Spyridon Alexis, Spyridon Vrakas, Constantina Argyropoulou, Vasileios Lazaris, Panagiota Spyropoulou, Vasiliki Labropoulou, Nicoletta Georgara, Maria Lykouresi, Marina Karakantza, Chrysoula Alepi, Argiris Symeonidis

**Affiliations:** ^1^Department of Transfusion Medicine and Blood Bank, “Tzaneion” General Hospital, Piraeus, Greece; ^2^Hematology Division, Department of Internal Medicine, University of Patras Medical School, Patras, Greece; ^3^Gastroenterology Department, “Tzaneion” General Hospital, Piraeus, Greece; ^4^Department of Transfusion Medicine and Blood Bank, University Regional General Hospital of Patras, Patras, Greece; ^5^Haematology and Transfusion Department, National Health Service Blood and Transplant, Leeds Teaching, Hospital Trust, Leeds, United Kingdom

**Keywords:** thalassemia, transfusion, adverse effects, microchimerism, immunomodulation

## Abstract

**Background:**

Transfusion-associated microchimerism implies the presence of allogeneic hematopoietic cells in an individual, following the transfusion of a blood product. It is a transfusion-related adverse effect/long-term consequence, which has not been well-investigated among regularly transfused patients with thalassemia.

**Patients and Methods:**

We investigated 64 regularly transfused, homozygous β-thalassemic patients and 21 never-transfused healthy volunteer blood donors (controls) for the presence of microchimerism in their sera, using real-time PCR targeting circulating allogeneic, both, Human Leukocyte Antigen-DR isotype (HLA-DR) and non-HLA alleles. The investigation was longitudinally repeated in patient subsets for more than 2 years. Results were correlated with clinical and laboratory parameters, peripheral blood lymphocyte immunophenotype, blood storage time, and donor's gender to identify potential contributing factors for microchimerism generation.

**Results:**

Overall, microchimerism was detected in 52 of the 64 patients (81.2%) and in 6 of the 21 controls (28.5%, *p* = 0.0001). Forty-four patients (68.7%) exhibited long-term microchimerism (persisted for more than 6 months), confirmed at all time-points investigated. Microchimerism was more frequent among elderly, women, splenectomized and more heavily transfused patients, and among those who exhibit higher serum ferritin levels. In these patients, a distinct descending pattern of CD16^dim^+CD56^dim^+ natural killer (NK)-cells (*p* < 0.001) and an ascending pattern of CD4+CD25^bright^CD127– regulatory T-cells (*p* = 0.022) for increasing allelic burden were noticed, suggesting the establishment of recipient immune tolerance against the donor-derived chimeric alleles. Both splenectomized and non-splenectomized thalassemic patients exhibited the same trend. The storage time of transfused blood products and donor/gender mismatch had no impact on the development of microchimerism.

**Discussion-Conclusive Remarks:**

Transfusion-associated microchimerism appears to be a very common complication among multi-transfused thalassemic patients. The potential clinical consequences of this phenomenon remain as yet unclear. Immune tolerance attributed to disease itself and to repeated transfusions might at least in part explain its appearance.

## Introduction

Beta (β)-thalassemias represent a group of hereditary disorders of hemoglobin synthesis, characterized by β-globin chain deficiency, either due to gene hypo-transcription, resulting from mutations of the promoter area, or from the production of a dysfunctional β-globin chain, leading to the formation of an unstable tetramer (1). The prevalence of β-thalassemia is higher across the Mediterranean countries, namely, Greece (2), where the frequency of β-thalassemia heterozygotes is estimated to be 6–8%, but in certain endemic areas may reach 15–20% (3). Patients with transfusion-dependent β-thalassemia (TDT) do not use their own erythropoiesis at all, have usually received their first transfusion course before the completion of their second year of life and require a lifelong regular red blood cell (RBC) transfusion program consisted of at least one transfusion course per month, to maintain adequate hemoglobin levels, adapted to the degree of their impaired erythropoiesis. Adequacy of RBC transfusions, coupled with an effective iron chelation regimen, represents the main therapeutic interventions for these patients, to secure normal growth and prevent end-organ complications. Moreover, many patients with non-transfusion-dependent thalassemia (NTDT) defined as those, who do not require a regular transfusion program, rely to a variable degree on their own erythropoiesis and they usually receive less than 6 transfusion courses per year, as well as those with sickle-cell/β-Thalassemia may transiently or permanently become transfusion-dependent at an unpredicted time point in their life (4, 5).

Although RBC transfusion is a routine clinical procedure for several decades, with low frequency of adverse effects, disguise some obscure and unpredicted long-term complications. Transfusion-associated complications have been well-recognized and characterized and are classified in two main categories: acute-occurring or delayed reactions and long-term consequences, such as alloimmunization, iron overload, graft vs. host disease, and microchimerism. For highly transfused patients with TDT, the risk of such complications is anticipated to be higher. The lastly mentioned long-term consequence, namely, transfusion-associated microchimerism (TA-MC) is not easily documented in routine clinical practice and has not yet been very well-characterized, particularly, in this patient population (6).

Transfusion of any blood product has been associated with the risk of long-term engraftment of donor-derived genetic material in the recipient, but the potential implications of this finding have not been studied sufficiently. Microchimerism in general is defined as the presence of a small percentage (3–5%) of allogeneic hemato/lymphopoietic cells in an individual, following the transfusion of a blood product, transplantation, or pregnancy (7–11). TA-MC was initially described in 1977, as the cytogenetic evidence of donor-derived leucocyte proliferation within 7 days following RBC transfusions (12). It has mainly been studied among trauma patients, its real frequency is unknown, but surprisingly, there is a paucity of data for multi-transfused patients with congenital or acquired blood disorders, requiring regular RBC transfusions. For trauma patients, who are transfused for a short period of time, it is also unknown why they develop, not only short-term (the existence of chimeric cells ≤ 6 months) but even long-term (the existence of chimeric cells >6 months) TA-MC (13–16) and which factors favor this complication (17).

This study aimed to investigate the frequency of TA-MC of all types (either short- or long-term) in a representative group of patients with thalassemic in Greece to detect and quantify microchimerism, assess its clinical significance and its consequences, and identify potentially existing predisposing factors for its appearance.

## Patients and Methods

### Study Population

Overall, 205 samples from 85 individuals were examined between March 2013 and May 2016. Sixty-four were thalassemic patients (target group, women *N* = 32, men *N* = 32, median age 42 years, range 22–60 years) and 21 were never-transfused male volunteer blood donors randomly selected during the study period (control group, median age 41 years, range 24–53 years, p:n.s.). Patients were tested 14–16 days following their previous RBC transfusion, whereas for controls, an extra ethylenediaminetetraacetic acid (EDTA) tube was collected, during the blood donation course. All patients with TDT received concentrated and leucocyte-depleted RBC (18), and all participated subjects were thoroughly informed about the study and signed informed consent. The study was approved by the Ethical and Scientific Committee of the Tzaneion Hospital (5th issue of the 17th Meeting of the Committee in June 13, 2012).

To investigate the duration of TA-MC existence, 57 of the 64 patients (89%) were re-tested after a median of 9.5 months, and among them, 21 non-completely transfusion-dependent (NTDT patients) were examined for a third time, following a median of 17.6 months from the second test and 26.4 months from the baseline one.

### Sample Processing

Peripheral blood samples were collected upon arrival to the Hospital, before the scheduled transfusion course, so that the last transfusion had been administered at least 14 days before sampling. Specimens were drawn in 5 ml EDTA tubes and were frozen immediately at −80°C. Samples were thawed for analysis at room temperature and blood lysates were prepared, using the PureLink Genomic DNA Mini Kit (Invitrogen, Thermo Fisher Scientific, USA). Lysates proceeded to DNA isolation and purification, using a spin column-based centrifugation. DNA was washed and eluted with 100 μl of Elution Buffer. DNA concentration was quantified by an ND-2000 spectrophotometer (NanoDrop, Thermo Scientific, USA).

### Microchimerism Assessment

**Figure 1:**
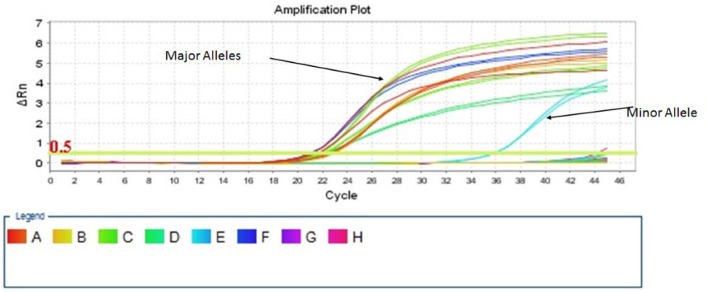
Distinguishing the major and the minor chimeric population, according to the threshold value (C_T_).

For TA-MC detection, two quantitative, allele-specific real-time PCR (QAS-PCR) assays were used, based in the work of Alizadeh et al. (19) and the adoption and optimization for Microchimerism detection from Lee et al. (20) They first detect minor leucocyte subpopulations, targeting allogeneic HLA-DR alleles, whereas the second works complementarily, detecting non-HLA loci on nine chromosomes, for naturally occurring insertion-deletion polymorphisms (S01–S011) (19, 20). Negative and positive controls for both panels were run in parallel with patients' samples. A mixture of 10 μl total PCR volume per well was needed, consisting of 5 μl *PowerUp* SYBR Green Master Mix (Applied Biosystems, Life Technologies, USA), 2 μl of Forward and Reverse primers and variable volumes of cDNA template and RNase-free water, depending on the DNA concentration of each sample, considering 100 ng/μl as the desirable DNA concentration. Pre-amplification procedures were carried out in a separate room from the amplification one, to avoid contamination. Real-time PCR was performed with the *StepOnePlus* System (Applied Biosystems, USA), using the following cycle conditions: 10 min at 95°C, followed by 45 cycles of 30 s at 95°C, 30 s at 64°C, and 45 s at 72°C. A Melt Curve Cycling stage was performed with 15 s at 95°C, 1 min at 60°C, and 15 s at 95°C, to assess the specificity of amplified products. Results were analyzed, using the Relative Quantification Method (ΔΔC_T_), with the *StepOne* Software v2.3 showing the ratio between the major and the minor populations (Life Technologies, USA). The patient's major TA-MC type was expected to exist in many DNA copies and cross the threshold value (C_T_) at a low cycle number. Conversely, the donor's minor type (microchimeric cells) was expected to have higher C_T_, making the two populations distinguishable, since fewer DNA copies would exist, and more replication cycles would be necessary to make them detectable (Figure 1). All PCRs were performed in duplicate and most positive samples were re-tested to exclude false-positive results. Microchimerism was defined by the appearance of minor alleles with a cycle threshold 8–10 cycles later than the major sequence pattern for the InDel panel. The major allelic population amplifies between 19 and 24 cycles while the minor population amplifies between 27 and 34 cycles (18). Respectively, for the HLA panel, the major alleles amplified on an early cycle 19 up to 26 and minor between 27 and 36. However, as have been previously reported, the concentration of the minor alleles can be extremely low after the first exposure and this kind of alleles increased over years, until stabilization to a level as much as 4% of all circulating white blood cells (WBCs) (20) This is the reason that every PCR signal cannot be ignored or characterized as noise but have to be carefully checked and retested. Positivity beyond the above C_T_, was characterized as low (C_T_ ≥ 33–36.9), very low (C_T_ ≥ 37–40.9), and borderline (C_T_ ≥ 41–42.9), when these results were confirmed by a new confirmatory PCR. This positivity is most likely to be attributed to different microchimerism levels, but rarely due to contamination. Contamination issues have been avoided by checking the three duplicated negative controls that “ran” in every PCR plate. Reproducibility for patients' major alleles, for two up to three different time points (by testing different samples), was also a verification for our results.

The sequences of primers used for the detection of HLA-DR alleles and of Insertion-Deletion Polymorphisms are shown in Tables 1, 2, respectively.

**Table 1:** Sequences of primers for the HLA-DR panel.

**Primer name**	**Forward**	**Reverse**
DR1	CTTGTGGCAGCTTAAGTTTGAATG	GGACTCCTCTTGGTTATAGATGCA
DR3	TACTTCCATAACCAGGAGGAGA	TGCAGTAGTTGTCCACCCG
DR4	GTTTCTGGAGCAGGTTAAAC	CCGCTGCACTGTGAAGCTCT
DR7	CCTGTGGCAGGGTAAGTATA	CCCGTAGTTGTGTCTGCACAC
DR8	AGTACTCTACGGGTGAGTGTT	CTGCAGTAGGTGTCCACCAG
DR9	CCCGTAGTTGTGTCTGCACAC	GTTCTCTGATGCAGGATAAGTTT
DR10	CGGTTGCTGGAAAGACGCG	CTGCACTGTGAAGCTCTCAC
DR11	GTTTCTTGGAGTACTCTACGTC	CTGGCTGTTCCAGTACTCCT
DR12	AGTACTCTACGGGTGAGTGTT	CACTGTGAAGCTCTCTCCACAG
DR13	TACTTCCATAACCAGGAGGAGA	CCCGCTCGTCTTCCAGGAT
DR15	CTGTGGCAGCCTAAGAGGGAGT	CCGCGCCTGCTCCAGGAT
DR16	CTGTGGCAGCCTAAGAGGGAGT	AGGTGTCCACCGCGGCG
DQA REFERENCE	GTGCTGCAGGTGTAAACTTGTACCAG	CACGGAAGCAGCGGTAGAGTTG

**Table 2:** Sequences of primers for the insertion-deletion polymorphisms panel.

**Primer name**	**Forward**	**Reverse**
S 01	GGTACCGGGTCTCCACATGA	GGGAAAGTCACTCACCCAAGG
S 02	GCTTCTCTGGTTGGAGTCACG	GCTTGCTGGCGGACCCT
SO-3	CTTTTGCTTTCTGTTTCTTAAGGGC	TCAATCTTTGGGCAGGTTGAA
SO-4	CTGGTGCCCACAGTTACGCT	AAGGATGCGTGACTGCTATGG
SO-4B	CAGTCACCCCGTGAAGTCCT	AGGATGCGTGACTGCTCCTC
SO-6	TGGTATTGGCTTTAAAATACTGGG	TTTCCCCCATCTGCCTATTG
SO-7	GGTATTGGCTTTAAAATACTCAACC	TGTACCCAAAACTCAGCTGCA
SO-7B	CTGGATGCCTCACTGATCCA	CAGCTGCAACAGTTATCAACGTT
SO-8	GCTGGATGCCTCACTGATGTT	TGGGAAGGATGCATATGATCTG
SO-8B	GGGCACCCGTGTGAGTTTT	TCAGCTTGTCTGCTTTCTGGAA
SO-9	TAGGATTCAACCCTGGAAGC	CCAGCATGCACCTGACTAACA
SO-11	GGACTGAGGCTCCCACCTTT	GCATGGACTGTGGTCTGCAA
GAPDH REFERENCE	GGTACCGGGTCTCCACATGA	GGGAAAGTCACTCACCCAAGG

### Flow Cytometry Analysis

Following an interim analysis of the obtained results from the first 18 patients, we decided to investigate the peripheral blood immunophenotype profile of our patients. Thus, a sub-cohort of 9 out of the 12 patients (75%) who did not exhibit TA-MC, and 42 of the 52 (81%), who exhibited TA-MC, were analyzed immunophenotypically at a median of 5.7 months (range 0–9 months) following the detection of TA-MC. In these 51 patients, there were 25 women and 26 men, and gender distribution was not significantly different between the two subgroups, according to the manifestation of TA-MC.

Peripheral blood lymphocyte subpopulations were estimated, using three 5-color protocols, formulated as shown in Table 3. The panel consisted of 12 monoclonal antibodies, targeting the basic T-helper (CD3+CD4+), cytotoxic (CD3+CD8^dim^+ and CD3+CD8^bright^), regulatory (CD3+CD4+CD25^bright^+CD127–), B (CD20+), natural killer (NK; CD3–CD16^bright^+ and/or CD56^bright^+CD8+/CD3–CD56^dim^CD16^dim^), NK/T (CD3+CD16+CD56+), and the NK/T subset CD3^bright^CD16+CD56+ (most probably T_γδ_), memory cells (CD45RO+), and lymphocytic middle (CD25+) and late (HLA-DR+) activation status. All monoclonal antibodies were purchased from Beckman Coulter, USA.

**Table 3:** Flow cytometry analysis panel used for the detection of various lymphocyte subpopulations.

**Color**	**Protocol 1: NK, NK-T, and CD8 cytotoxic**	**Protocol 2: T-cells, T-reg, and activation status**	**Protocol 3: Memory, B cells, and monocytes**
FITC	CD3	CD127	CD45RO
PE	CD16+CD56	CD25	CD14
ECD	CD8	HLA DR	CD8
PC5	CD4	CD4	CD20
PC7	CD45	CD3	CD45

### Sample Preparation

Sample preparation was conducted within 24 h of acquisition. A mixture of 75 μl of whole peripheral blood with the monoclonal antibodies, at a volume of 13 μl for fluorescein isothiocyanate (FITC) and phycoerythrin (PE) conjugates and 7 μl for ECD, PC5, and PC7 conjugates, respectively, was prepared and incubated for 15 min at room temperature, protected from light. A 1.5 ml of BD Pharm Lyse diluted to 1 × concentration was used to lyse RBC, following antibody staining, at a final volume of 1.5 ml for 15 min. Flow cytometry sample acquisition was performed immediately after sample preparation, using an FC 500 Cytometer (Beckman Coulter, USA).

### Statistical Analysis

Comparisons of parametric data were made using Student's *t*-test or one-way ANOVA for variables with normal distribution. For non-parametric data, the Mann–Whitney *U*-test was used to compare differences between two groups, and Kruskal-Wallis and independent samples median tests for comparisons between more than two groups were used. A *p* of < 0.05 was considered statistically significant. Correlations between the number of chimeric alleles and percentages of lymphocyte subsets were tested with the Spearman's R. Analysis and graphs were made with Microsoft Excel 2010 and Stata 9.0 and with the SPSS Statistical package version 25. The Flow Cytometry results were analyzed using the Kaluza software version 2.1.

## Results

### Detection of Microchimerism

**Figure 2:**
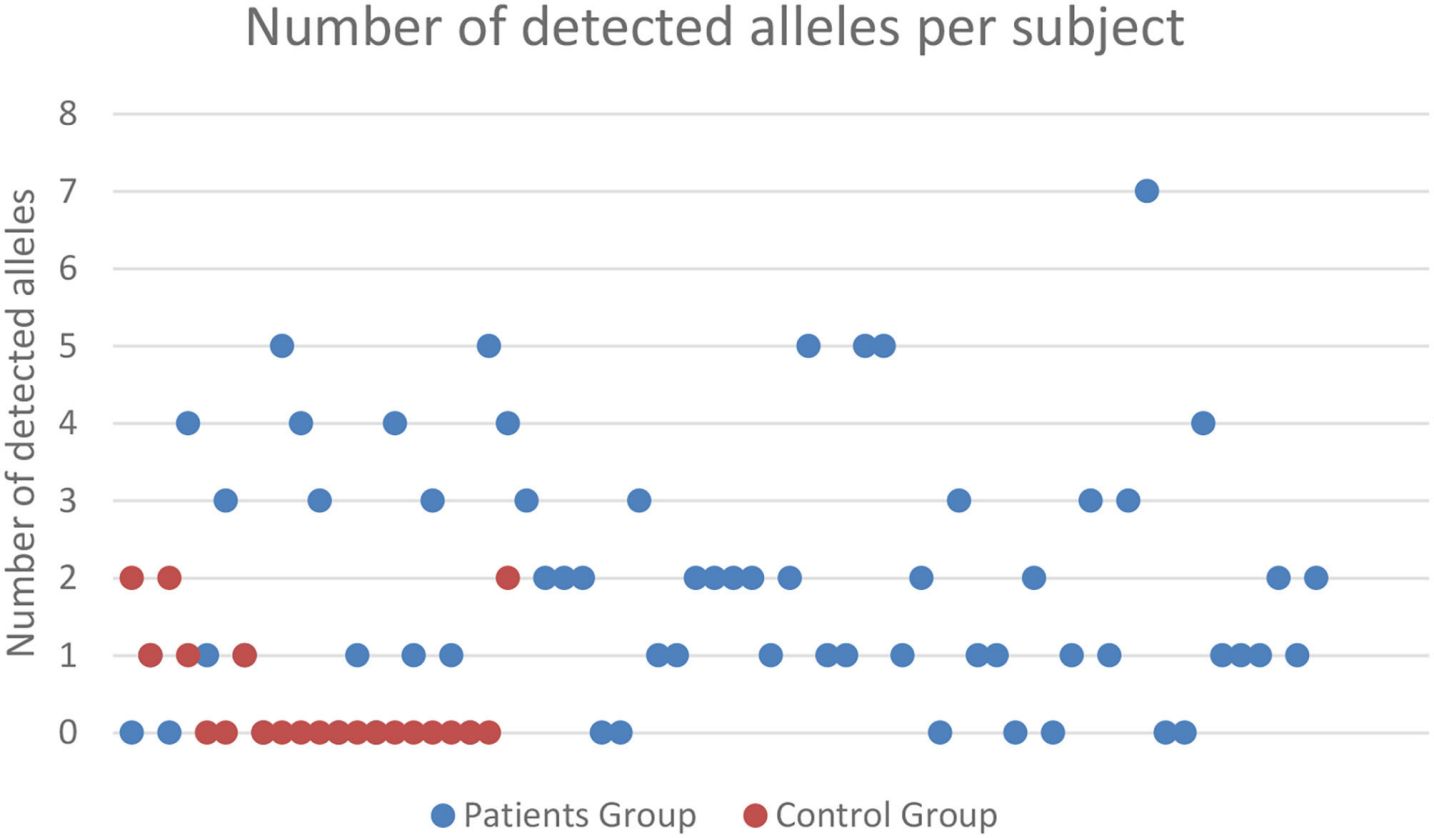
Frequency and severity of microchimerism (number of chimeric alleles detected) according to the subject group tested. Only patients with TDT exhibited ≥3 positive chimeric alleles, and there was a big difference in the proportion of subjects exhibiting 1 or 2 chimeric alleles, between TDT patients and controls. TDT, transfusion-dependent β-thalassemia.

**Figure 3:**
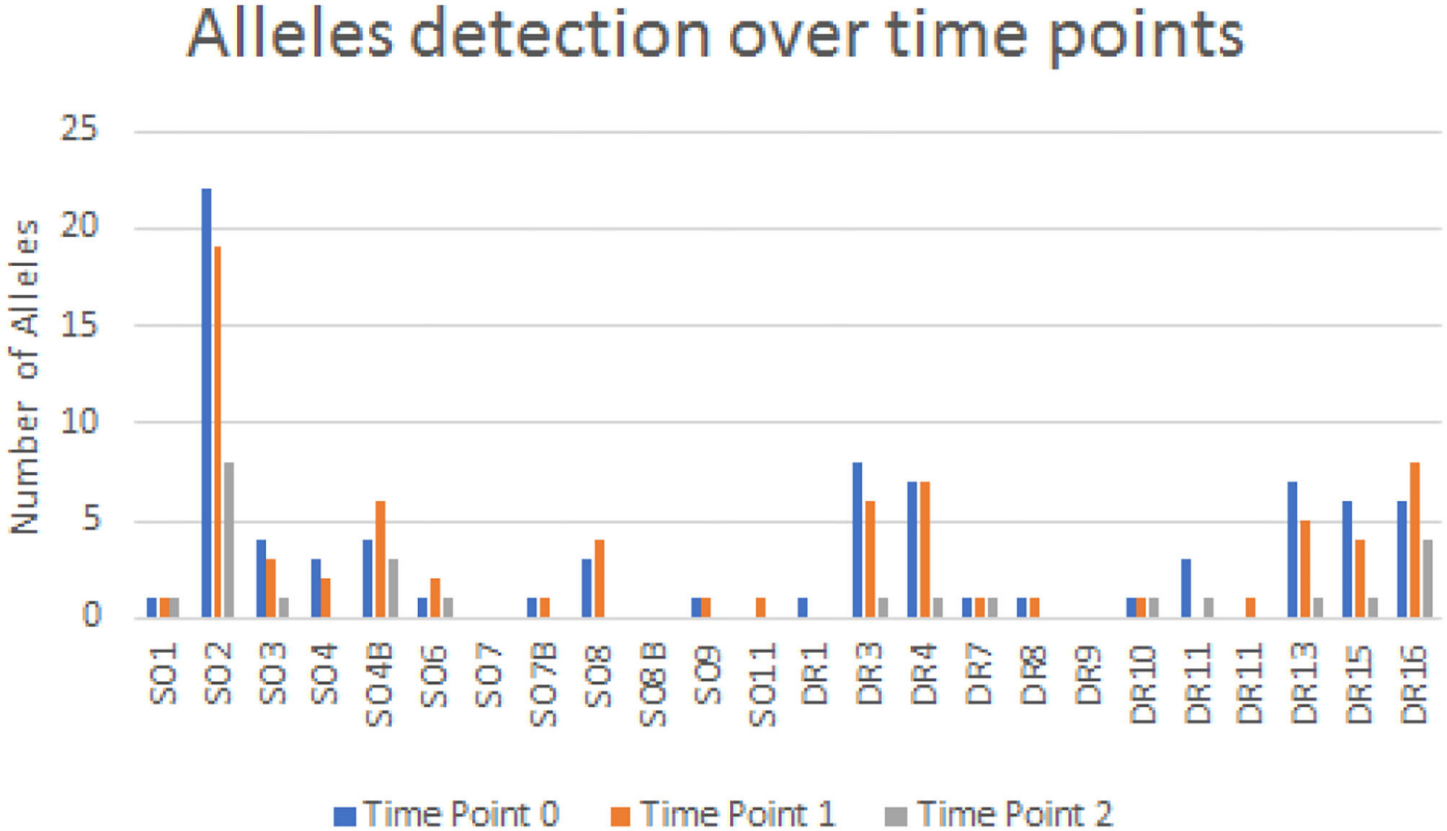
Descriptive diagram for allele detection over time.

Microchimerism was detected in both groups but was significantly more prominent in the patient group (52/64 ≈ 81.2%) compared to the control group (6/21 ≈ 28.5%, *p* = 0.0001; Figure 2). The detection of chimeric alleles among the controls could be interpreted in the context of persistent maternal microchimerism, as has previously described (10, 21–24). The quantification of the detected microchimerism, in terms of evaluating the number of chimeric alleles and the duration of the phenomenon, showed that the number of chimeric alleles in the control group did not exceed two, whereas in the patient group it reached up to seven chimeric alleles per patient tested (Figure 2). Twenty-two out of 64 patients (34.4%) had evidence of long-term TA-MC, 8 (12.5%) patients of only short-term, whereas the remaining 22 (34.4%) exhibited both short- and long-term TA-MC. In 7 (33.3%), among the 21 patients with NTDT, for whom microchimerism was confirmed at three time points, the gradual appearance of six new, but also the disappearance of four previously existing short-term chimeric polymorphisms, during a median period of 26.4 months was observed (Figure 3). No control subject was retested as the interpretation for them was a chronically existing maternal microchimerism (10, 21–24).

### Association of TA-MC With Potential Contributing Factors

**Figure 4:**
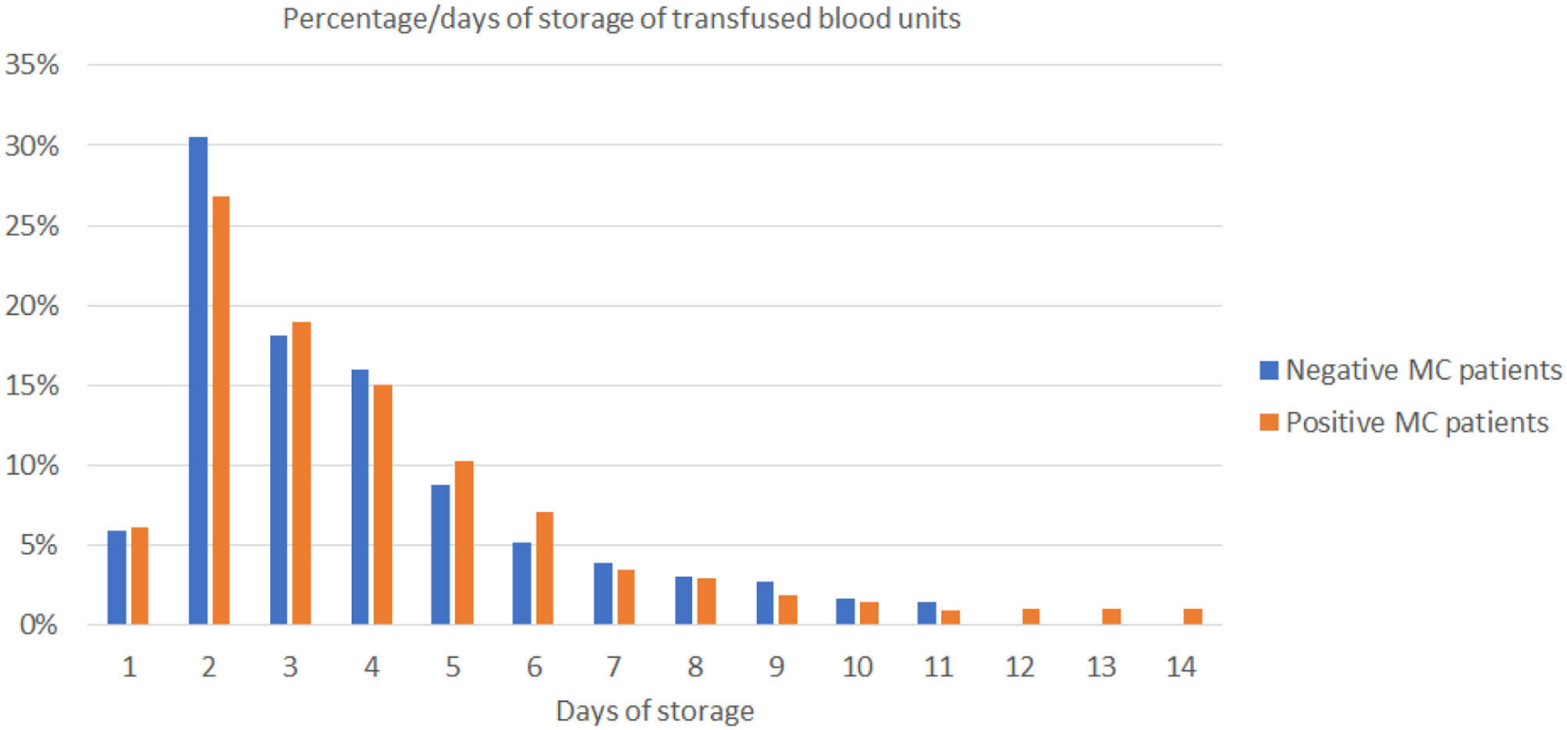
The diagram shows the range of storage days of the 1,241 blood units that had been transfused on 11 non-MC (blue bars) and 1,173 blood units on 14 MC patients (orange bars). MC, microchimerism.

To identify potential contributing factors that might influence TA-MC development among thalassemic patients, we investigated the relationship of detected TA-MC with patients' clinical features. Gender (*p* < 0.004), age (*p* = 0.021), transfusion frequency (low: ≤ 6, intermediate: 6–20, high: >20 transfused RBC units/year; *p* = 0.043), and serum ferritin levels (*p* = 0.041) were significantly correlated with the number of chimeric alleles detected (Table 4). Moreover, previous splenectomy (*p* = 0.054) and the presence of autoimmune disorders (*p* = 0.052) showed an association of borderline significance. We furthermore examined parameters related to the transfusion process, such as the storage time of RBC units and donor's gender. Totally, 2,414 RBC units transfused over a median period of 44 months were examined. For 11 patients without established TA-MC. the median storage time of transfused units was 4.54 days and 75% of the donors were men. Similarly, for 14 patients exhibiting the highest number of chimeric alleles, the median storage time was 4.24 days and 74.5% of the donors were men (p:n.s) (Figure 4). Thus, no association between these two parameters and microchimerism establishment was proved. Gestation was also investigated as a potential risk factor for microchimerism development, in combination with the transfusion therapy of the thalassemic women, but again, no significant association was found.

**Table 4:** Patient demographics and main clinical and laboratory characteristics associated with TA-MC status.

**Characteristics of 64 subjects**	**Number and percentage (%) of microchimeric**	**Average of chimeric alleles ±standard deviation**	***P***
**Sex**			
Male (32)	22 (68.7%)	1.15 ± 0.97	**0.0004**
Female (32)	30 (93.7%)	2.46 ± 1.69	
**Age**			
Median: 33.6 (31)	24 (77.4 %)	1.35 ± 1.00	**0.021**
Median: 52.5 (33)	28 (84.8%)	2.24 ± 1.79	
**Transfusion frequency**			
High (38)	32 (84.2%)	1.89 ± 1.47	**0.040**
Intermediate (16)	14 (87.5%)	2.19 ± 1.78	
Low (10)	6 (60%)	0.90 ± 0.83	
**Splenectomized**			
No (28)	22 (78.5%)	1.39 ± 1.01	0.054
Yes (36)	30 (83.3%)	2.13 ± 1.76	
**Autoimmune disorder**			
No (60)	48 (80%)	1.71 ± 1.46	0.052
Yes (4)	4 (100%)	3.25 ± 1.78	
**Pregnancy**			
No (24)	22 (91.6%)	2.41 ± 1.70	0.712
Yes (8)	8 (100%)	2.62 ± 1.65	
**Endocrinal disorders**			
No (9)	9 (100%)	1.77 ± 1.22	0.906
Yes (55)	43 (78.1%)	1.81 ± 1.57	
**Cardiac disorders**			
No (43)	37 (86%)	1.81 ± 1.35	0.921
Yes (21)	15 (71.4%)	1.80 ± 1.84	
**HCV infection**			
No (41)	36 (87.8%)	2.02 ± 1.45	0.098
Yes (23)	16 (69.5%)	1.43 ± 1.58	
**Past HBV infection**			
No (37)	30 (81%)	1.86 ± 1.59	0.734
Yes (27)	22 (81.4%)	1.74 ± 1.42	
**Lymphocytes total number** ^*^			
(1,000–2,321)	25 (83.3%)	1.80 ± 1.4	0.982
(2,419–7,200)	22 (81.4%)	1.79 ± 1.7	
**Γ-globulins total amount** ^**^			
(533–1,490)	27 (77.1%)	1.94 ± 1.68	0.437
(1,500–2,744)	24 (85.7%)	1.64 ± 1.31	
**Ferritin median value**			
(85–4,667)	49 (85.9%)	1.94 ± 1.53	**0.041**
(5,049–7,641)	3 (42.8%)	0.71 ± 0.88	

*Female patients of more advanced age, more heavily transfused with higher serum ferritin levels were associated with stronger and longer-standing microchimerism. TA-MC, transfusion-associated microchimerism*.

* *Data was available for 59 patients*.

** *Data was available for 63 patients. Statistically significant results are depicted in bold*.

### Flow Cytometry Analysis

The possibility that underlying immune dysregulation might influence TA-MC development or even the reverse (25), meaning that TA-MC might affect immune homeostasis, prompted us to investigate the peripheral blood immunological profile of these patients. The major T-cell subpopulations (CD3+, CD4+, and CD8+), their memory subsets (CD45RO+), B cells (CD20+), and their activation status (assessed by HLA-DR and CD25 expression on the CD3+ and CD4+ lymphocytes) did not exhibit any significant difference when compared between patients with ascending number of chimeric alleles (Table 5). However, a significant correlation between the number of detected chimeric alleles and the proportion of CD3+CD25^bright^+ (a population in which regulatory T-cells are included) was found (Table 5). Further investigation revealed significant alterations, proportional to the microchimerism grade, for selected lymphocyte subpopulations with known immunoregulatory properties, such as NK, NK/T, and regulatory T-cells. Table 5 displays all results of the lymphocyte subpopulations, examined across patients' groups, as defined by the number of chimeric alleles detected by PCR and their statistical significance. Displayed results are the following: the Spearman correlation; either positive or negative, suggesting a potential favorable/unfavorable/independent role that each population holds, regarding the establishment and the grade of microchimerism detected, and the independent samples Kruskal–Wallis and median tests statistical significance, depicting the differences in distributions and medians of the populations across patient groups with different chimeric alleles.

**Table 5:** Results for the major lymphocyte subpopulations, examined across patients' groups, defined by the number of chimeric alleles detected by PCR.

**Patient groups defined by the number of chimeric alleles detected**		**Spearman's Rho**	
**Major lymphocyte subpopulations**	***N***	**Correlation coefficient**	**Significance (2-tailed)**	**Kruskal–Wallis test**	**Median test**
CD20+ (B cells)	51	−0.033	0.817	0.955	0.998
CD3+ (total T-cells)	51	−0.034	0.811	0.882	0.649
CD3– (non T-cells)	51	0.034	0.811	0.882	0.445
CD8+CD3+ (CD8 T-cells)	51	−0.227	0.109	0.516	0.365
CD4+CD3+ (CD4 T-cells)	51	0.142	0.319	0.374	0.081
CD4+CD25brCD127– (T-reg)	51	**0.321**	**0.022**	**0.170**	**0.012**
**Activation status**				
HLADR^dim^	45	−0.088	0.567	0.860	0.200
HLADR^bright^	45	−0.010	0.947	0.417	0.445
CD3+CD25^bright^	45	**0.339**	**0.023**	0.620	0.111
CD3+CD25^dim^	45	0.244	0.106	0.468	0.342
CD3+HLADR^bright^	45	0.201	0.185	0.117	0.279
CD3+HLADR^dim^	51	0.116	0.417	0.616	0.573
**Memory cells**				
CD45RO+ (memory cells)	51	0.195	0.170	0.636	0.373
CD4+CD45RO+ (CD4 memory)	51	0.158	0.267	0.371	0.573
CD8+CD45RO+ (CD8 memory)	51	0.223	0.115	0.270	0.432
**NK cell populations**				
CD3+CD16+CD56+ (NK/T)	51	**−0.280**	**0.046**	**0.021**	**0.020**
CD3^bright^CD16+CD56+ (NK/Tγδ)	51	0.131	0.358	0.291	0.487
CD3–CD16^dim^CD56^dim^ (CD16^dim^CD56^dim^ NK)	51	**−0.435**	**0.001**	**0.032**	**0.049**
CD3–CD16^bright^CD56^bright^ (CD16^bright^CD56^bright^ NK)	51	0.184	0.196	0.798	0.903

*The table summarizes the Spearman correlation (either positive or negative) of each population assessed with the number of chimeric alleles detected and the differences in medians and distributions of the populations across the same patient groups (median test, Kruskal–Wallis test). Statistically significant results are depicted in bold. Mature NK cells and NK/T cells were significantly reduced, whereas regulatory T-cells were significantly increased, and these populations, and as activated T-cells expressing CD25^bright^ were significantly associated with higher number of detected chimeric alleles in patients' peripheral blood*.

The same investigational approach was then applied for sub-cohorts of patients, characterized by their disease subtype and splenectomy status (transfusion dependent vs. non-transfusion dependent and splenectomized vs. non-splenectomized) in order to examine the effect of these major factors on the immunological profile of the patients. These findings are discussed below.

### NK-Cells

**Figure 5:**
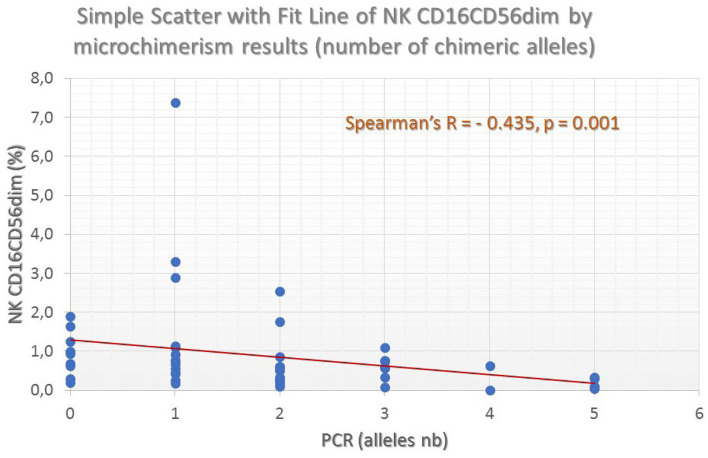
Fluctuation of mature NK-cell frequencies (as defined by the CD16^dim^CD56^dim^ co-expression profile) by the number of chimeric alleles detected in the thalassemic patient population. A clear and significant inverse association can be seen.

**Figure 6:**
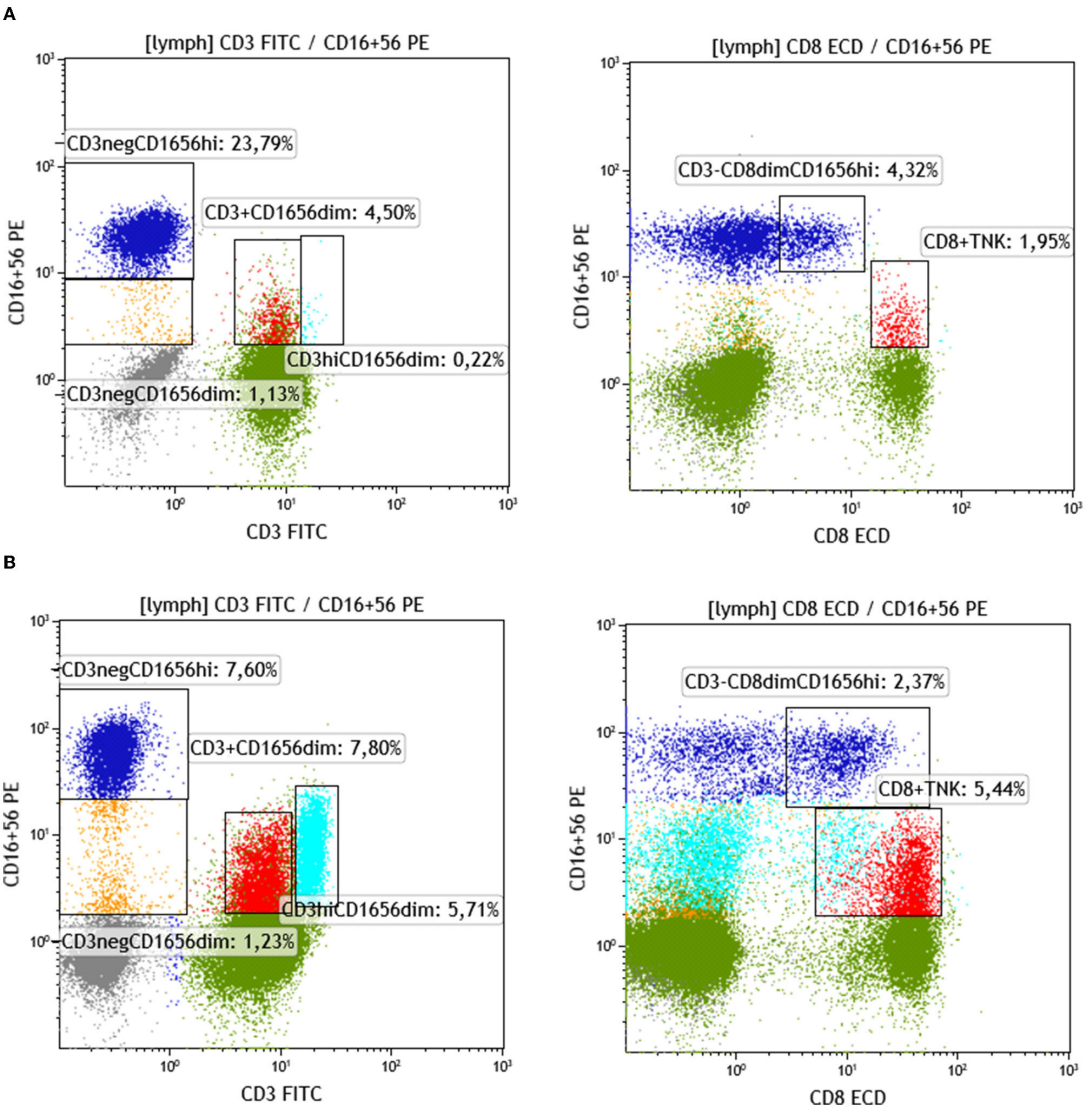
Identification and quantification of NK and NKT in patients with a different allelic burden. **(A)** Patient with no chimeric alleles detected. **(B)** Patient with three chimeric alleles detected (splenectomized) (green: CD3+ non-NK, gray: CD3– non-NK, blue: NK CD56CD16^br^, red: CD8+NKT, turquoise: CD3^br^ T/NK yellow: NK CD56CD16^dim^).

The following NK-cell subsets were examined: CD16^dim^+CD56^dim^+ and CD16^bright^+CD56^bright^+ (classical NK-cells), NK/T cells, and NK/T cells with CD3^high^ expression (probably NK/T_γδ_). Median proportions of NK-cell subsets across patient groups exhibiting a different number of chimeric alleles were then compared. The percentage of CD3–CD16^dim^+CD56^dim^+ cells showed a statistically significant difference between the 6 patient groups, exhibiting a descending pattern, as the detected chimeric allelic burden was increasing (Kruskal–Wallis *p* = 0.032; Figure 5). NK/T-cells (CD3+CD16+CD56+) also exhibited a statistically significant difference, but with a less prominent proportional relationship with the number of chimeric alleles. The major NK-cell population (CD3–CD16^bright^+CD56^bright^+) did not show any significant difference. Results of the investigated NK-cell subpopulations are presented in Table 5 and comparative flow cytometry analysis (plots) from two patients, one not exhibiting microchimerism vs. another with high allelic burden, is depicted in Figure 6.

### Regulatory T-Cells

**Figure 7:**
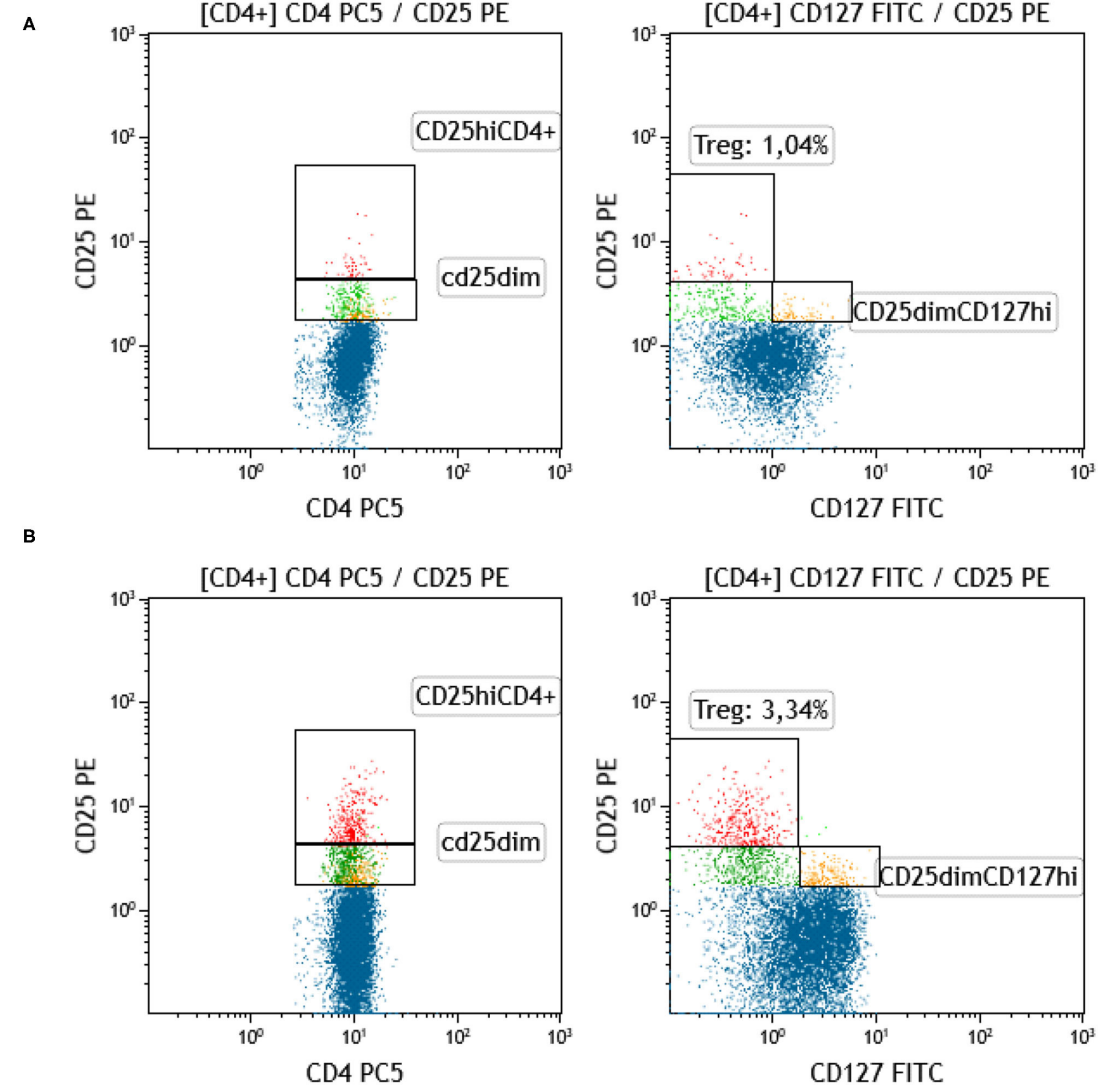
Identification and quantification of T-reg in patients with a different allelic burden. **(A)** Patient with no chimeric alleles detected. **(B)** Patient with five chimeric alleles detected (red: T-reg [CD4+CD25^hi^CD127–], blue: CD4+CD25–, green: CD4+CD25^dim^CD127–, yellow: CD4+CD25^dim^CD127+).

**Figure 8:**
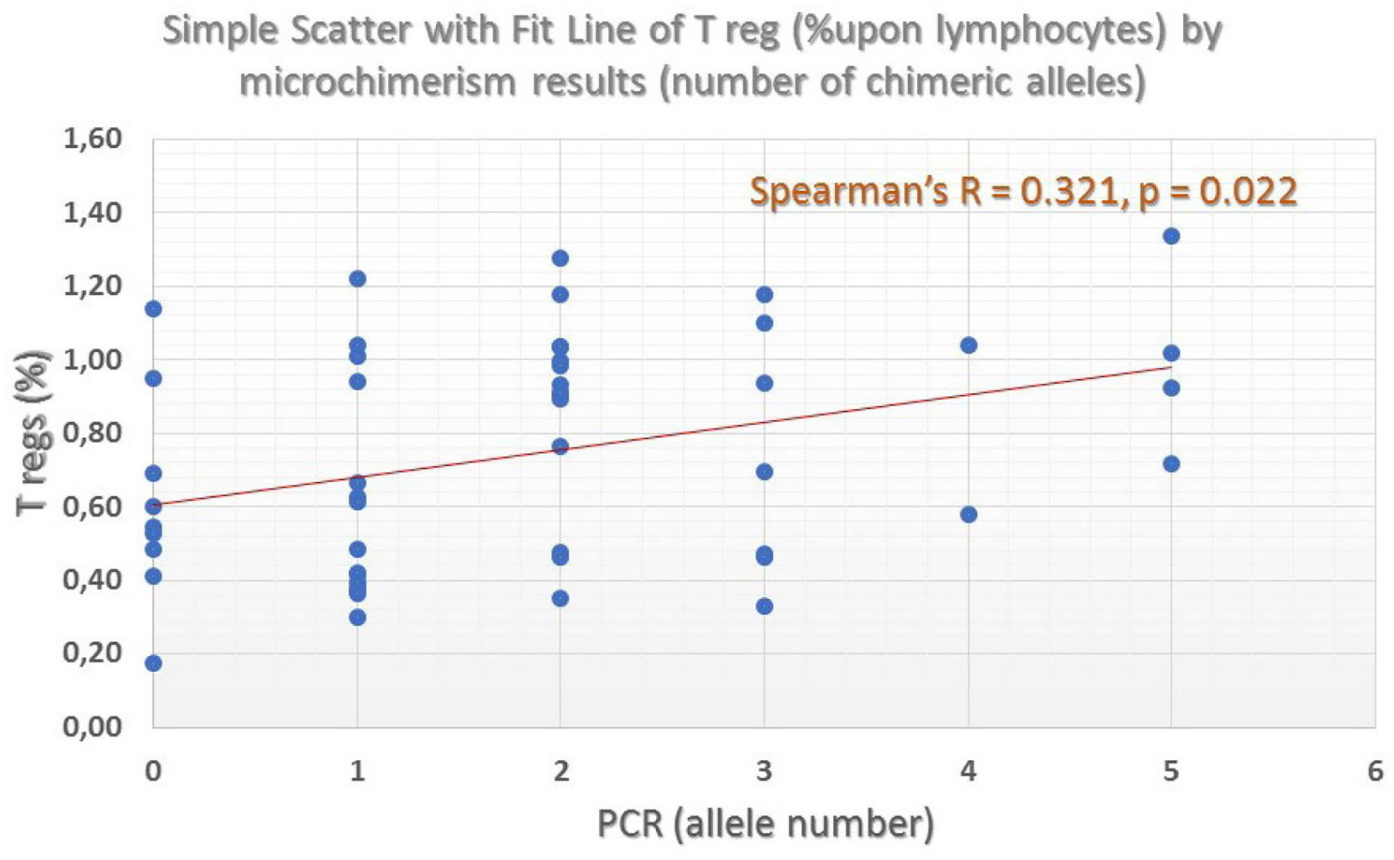
Percentage of Regulatory T-cells across different patient groups defined by the number of chimeric alleles. A clear ascending trend, in parallel with the number of detected chimeric alleles was found (grand median = 0.69, median test significance *p* = 0.012).

Regulatory T-cells were identified as the CD127 negative/dim subset of CD4+CD25 bright T-cells (CD3+) (as shown in Figure 7). Intracellular staining of transcriptional factor FoxP3 was omitted since CD127 is an adequate surrogate marker for T-reg identification (26) for a broad-analysis spectrum panel for lymphocytic subpopulations, as the one we developed for this study with the further advantage of making it more time- and cost-effective with the prospect of analyzing as many samples as possible. Independent sample median test showed statistically significant difference for the regulatory T-cell proportions, among patients with a different allelic burden (*p* = 0.012, Figure 8). Patients without TA-MC or those with a low number of detected chimeric alleles had the lowest percentages, whereas the subset expanded as the number of alleles increased. Examples of T-reg identification and enumeration in two patients with different chimeric allelic burden are displayed in Figure 7.

### Analysis of Patient Sub-Cohorts

Non-transfusion-dependent thalassemia patients exhibited a distinct ascending CD4+ lymphocyte pattern, as the number of chimeric alleles was increased (independent samples median test *p* = 0.056), however, the same was not true for all patients with TDT. Among previously splenectomized patients with NTDT (*N* = 11), those exhibiting TA-MC with ≥2 chimeric alleles (*N* = 4) had higher (although not significantly) percentage of CD4+CD45RO+ memory T-cells, the reverse pattern of decreased CD16^dim^+CD56^dim^ NK-cells (0.29 vs. 0.76%, independent samples median test *p* = 0.26, Mann–Whitney test *p* = 0.007), and increased CD16^bright^ mature NK-cells, when compared to the remaining seven, who exhibited <2 chimeric alleles (17.9 vs. 13.0%, independent samples median test *p* = 0.26, Mann–Whitney test *p* = 0.004).

Splenectomized patients with TDT (*N* = 14, 5 with >2 chimeric alleles, 9 with ≤ 2) exhibited also similar results (CD16^dim^+CD56^dim^+ cells: 0.54 vs. 1.25%, independent samples median test *p* = 0.54, Mann–Whitney test, *p* = 0.10). Non-splenectomized TDT patients (*N* = 24:20 with ≤ 2 alleles, 4 with >2 chimeric alleles) exhibited slightly increased regulatory T-cells, (0.76 vs. 0.68%) and CD56^dim^+ cells (0.58 vs. 0.34%) and decreased T/NK cells (4.12 vs. 5.80%) and memory T-cells (34.3 vs. 40.6%) as the number of the microchimeric alleles increased to >2. However, due to the small number of non-splenectomized patients with NTDT (*N* = 2), all differences were not statistically significant hence we did not perform further investigation (10, 18–20, 21–24, 26–28, 29, 30).

## Discussion

Microchimerism represents a complicated phenomenon, and several mechanisms are involved in its establishment. Moreover, many questions, related to the factors affecting its development and persistence for shorter or longer periods, among subjects exposed to similar predisposing conditions, still remain unanswered. Two important findings have emerged from this study. First, we have confirmed that patients with TDT, receiving lifelong RBC transfusions, commonly develop TA-MC even when transfused units are leuco-reduced, and second, we have demonstrated that TA-MC is associated with immune changes in the T-regs and some NK-cell subsets. Whether leuco-reduction is protective is contradictory, according to the published relevant information in other patient populations (18–20, 23–25). We have not approached this issue, since in our Hospital all immunocompromised, TDT, and other multi-transfused patients only receive leuco-reduced blood products, according to the European Council guidelines (the leukocyte content in the blood components we used is 1 × 10^6^ per unit, i.e., 95% CI). In our study, the high rate of microchimerism detected among healthy controls was somewhat surprising, however, similar results have also been obtained from previous studies and are interpreted in the context of persistent maternal microchimerism (10, 21–24). A small proportion might also be considered false positive, due to a high sensitivity of the PCR reagents we used. All control subjects tested, continue to donate blood and during the subsequent two to four years they did not report any problem of their health. However, the observed high frequency of TA-MC among thalassemic patients, and particularly, the high number of detected alleles can only be attributed to RBC transfusions. The obtained significant association between the number of detected chimeric alleles and serum ferritin levels, reflecting transfusion burden, supports this notion. Irradiation appears to significantly reduce the risk of TA-MC (27). Previous studies, mainly performed on trauma patients, have demonstrated that storage time was a predictor for TA-MC development. We were unable to confirm this finding. A potential explanation might be that different pathogenetic mechanisms that drive TA-MC generation in patients with TDT, compared to those occurring in trauma patients, and specific immunomodulatory conditions, attributed to the disease itself and/or to the chronic transfusion program, are developed in patients with TDT, favoring the generation of microchimerism.

Microchimerism reflects the presence of donor-derived antigenic and/or genetic material that evades immunologic surveillance of the recipient, most probably through the induction of tolerance. Immune tolerance might both, pre-exist and also, be induced or exacerbated by the repeated RBC transfusions. The persistence of donor's derived allogeneic lymphocytes or even diluted allogeneic HLA-antigens in the recipient's blood is both, favored by pre-existing immune tolerance, and induces further tolerogenic changes. In the majority of cases, this immunomodulation is incapable to induce transfusion-associated Graft versus Host Disease (GvHD), because the latter requires a larger quantity of donor lymphocytes, which must survive the recipient's cytotoxic reaction, recognize alloantigens, and orchestrate an immune response against the donor. When transfused lymphocytes are few and repeatedly administered, a type-II immune response might be induced, induced by the multiple antigenic stimuli, leading to immune tolerance. Immune tolerance increases the residence time of allogeneic cells in the recipient's blood and generates TA-MC. Type-II immune response might be stronger in cases of long-term microchimerism, as it has been shown in a well-designed prospective randomized French study on 37 cancer patients, on whom TA-MC was associated with a clear immunomodulation toward Th2 direction (28).

In our study, TDT patients with higher chimeric allelic burden exhibited a distinct immunologic profile, characterized by CD4+ T-lymphocyte (such as T-regs) expansion and by decreased NK-cell subsets. These findings reflect the existence of immune tolerance, which allows or even favors TA-MC development, through the immunomodulatory/immunosuppressive functions of T-regs and the lack of certain cytolytic effects of NK cells. The differences observed in various NK-cell subsets, especially among splenectomized patients, would be difficult to assess, considering the well-known persistent post-splenectomy NK lymphocytosis. Splenectomy might play an additional role in the expansion of the phenomenon since splenectomized patients usually exhibit higher percentages of T-regs and higher microchimeric allelic burden.

Immune tolerance, resulting from potential donor/recipient genotype relationship, should also be taken into consideration, especially in “closed” populations, living in small geographic areas, where blood donors and recipients may retain probabilities to be distantly related. To this point, the increased number of repeated detection of 7 alleles out of the 24 tested [S02, S04B, DR3, DR4, DR13, DR15, and DR16] (Figure 3), may partially be attributed to this explanation. Investigation of larger cohorts of multi-transfused patients and untransfused controls may clarify potentially existing predisposing factors for TA-MC development and for its prognostic significance. Clinically speaking, the previous γ-irradiation of all blood products and compliance with updated blood transfusion management protocols could be recommended, to avoid potentially as yet unknown consequences of microchimerism.

## Conclusion

The majority of multi-transfused thalassemic patients exhibit TA-MC in their peripheral blood. Risk factors for the establishment of microchimerism in this patient population are gender, age, and transfusion frequency. NK-T cell increase, usually post-splenectomy, but also T-reg imbalance, appears to influence TA-MC development and perpetuation, favoring the establishment of immune tolerance against allogeneic cells. Leucodepletion and blood storage time do not influence the appearance and duration of TA-MC.

## Limitations of This Study

This study aimed to demonstrate the presence of both, short and long-term TA-MC among multitransfused patients with β-thalassemia, since there was no information about this late consequence in the thalassemic patient population. It has not focused on the investigation of the presence of maternal microchimerism among the normal control subjects. Thus, controls have been tested only once and not longitudinally, to establish the frequency of existing long-term MC, relying on the results of previous studies. Moreover, this study has not investigated potential correlation of TA-MC with any clinical feature in the course and evolution of β-thalassemic patients, such as frequency of transfusion-induced alloimmunization, or the manifestation of autoimmune and neoplastic diseases at long-term follow up. Although we did not find an obvious correlation with any clinical parameter or feature of the patients during the study period, this issue requires further and in depth clinical investigation, which will be the aim of another study.

## Data Availability Statement

The original contributions presented in the study are publicly available. This data can be found here: https://doi.org/10.6084/m9.figshare.14646657.

## Ethics Statement

The studies involving human participants were reviewed and approved by Tzaneion Hospital Ethics and Scientific Committee, 5th issue of the 17th Meeting of the Committee in 13.6.2012. The patients/participants provided their written informed consent to participate in this study.

## Author Contributions

SM, MK, and AS designed the project. SM also performed PCR experiments, analyzed data, and co-wrote the paper. EV designed and performed Flow Cytometry experiments, analyzed data, and co-wrote the paper. AK supervised and designed samples collection and patient's data collection. SA performed Flow Cytometry experiments. SV performed the statistical analysis. CAr performed samples collection and preservation. PS supervised and co-designed the PCR experiments. VLaz and VLab offered the clinical patient care and retrieved clinical data. NG collected blood products' storage time and gender data. ML performed the samples and the primers data preparation. CAl supervised the research. AS supervised the research and co-wrote the paper. All authors contributed to the article and approved the submitted version.

## Funding

This research was supported by the research grants from the University of Patras.

## Conflict of Interest

The authors declare that the research was conducted in the absence of any commercial or financial relationships that could be construed as a potential conflict of interest.

## Publisher's Note

All claims expressed in this article are solely those of the authors and do not necessarily represent those of their affiliated organizations, or those of the publisher, the editors and the reviewers. Any product that may be evaluated in this article, or claim that may be made by its manufacturer, is not guaranteed or endorsed by the publisher.
